# miREC: a database of miRNAs involved in the development of endometrial cancer

**DOI:** 10.1186/s13104-015-1052-9

**Published:** 2015-03-28

**Authors:** Benjamin Ulfenborg, Sanja Jurcevic, Angelica Lindlöf, Karin Klinga-Levan, Björn Olsson

**Affiliations:** Systems Biology Research Centre – Bioinformatics, University of Skövde, Box 408, 541 28 Skövde, Sweden; Systems Biology Research Centre – Tumor Biology, University of Skövde, Box 408, 54128 Skövde, Sweden

**Keywords:** Endometrial cancer, MicroRNA, Database

## Abstract

**Background:**

Endometrial cancer (EC) is the most frequently diagnosed gynecological malignancy and the fourth most common cancer diagnosis overall among women. As with many other forms of cancer, it has been shown that certain miRNAs are differentially expressed in EC and these miRNAs are believed to play important roles as regulators of processes involved in the development of the disease. With the rapidly growing number of studies of miRNA expression in EC, there is a need to organize the data, combine the findings from experimental studies of EC with information from various miRNA databases, and make the integrated information easily accessible for the EC research community.

**Findings:**

The miREC database is an organized collection of data and information about miRNAs shown to be differentially expressed in EC. The database can be used to map connections between miRNAs and their target genes in order to identify specific miRNAs that are potentially important for the development of EC. The aim of the miREC database is to integrate all available information about miRNAs and target genes involved in the development of endometrial cancer, and to provide a comprehensive, up-to-date, and easily accessible source of knowledge regarding the role of miRNAs in the development of EC. Database URL: http://www.mirecdb.org.

**Conclusions:**

Several databases have been published that store information about all miRNA targets that have been predicted or experimentally verified to date. It would be a time-consuming task to navigate between these different data sources and literature to gather information about a specific disease, such as endometrial cancer. The miREC database is a specialized data repository that, in addition to miRNA target information, keeps track of the differential expression of genes and miRNAs potentially involved in endometrial cancer development. By providing flexible search functions it becomes easy to search for EC-associated genes and miRNAs from different starting points, such as differential expression and genomic loci (based on genomic aberrations).

**Electronic supplementary material:**

The online version of this article (doi:10.1186/s13104-015-1052-9) contains supplementary material, which is available to authorized users.

## Findings

### Background

Endometrial cancer is the most common cancer of the reproductive tract in women in developed countries. According to the World Cancer Research Fund (WCRF) around 288 000 women worldwide develop endometrial cancer annually and 74 000 died from this cancer in 2008 [1]. It has been shown in several studies that a number of microRNAs (miRNAs) are differentially expressed in EC tumors compared to healthy endometrial tissue [2-9]. MiRNAs are small RNA molecules that regulate gene expression by inhibition of translation or by degradation of their target mRNAs [10-12]. It has been shown that miRNAs play key roles in biological processes of relevance for cancer, such as development, differentiation, cell proliferation, apoptosis and stress response [13], and it has been estimated that up to 30% of human genes are regulated by miRNAs [14]. Distinct expression signatures have been found for practically every form of cancer in which miRNA expression has been studied [14].

Several studies have identified miRNAs, which are differentially expressed in EC compared to healthy endometrial tissue. For example, miR-205 was found to be over-expressed in EC by a number of authors [2,4-6] and has also been described to be upregulated in bladder and ovarian cancers [15,16], but downregulated in breast cancer [17]. The miR-200 family consists of five members (miR-200a, 200b, 200c, 141 and 429), which have all been shown to be upregulated in endometrial cancer [5,9,18]. Furthermore, the members of the miR-200 family together with miR-205 regulate the expression of target genes ZEB1 and ZEB2, which have been implicated to be involved in epithelial to mesenchymal transition and tumor progression [19]. Previous studies have also shown that members of the miR-96 cluster (hsa-miR-96, hsa-miR-182 and hsa-miR-183) are upregulated in endometrial cancer [5,6,18,20]. More recently, the downregulation of miR-199a-3p in EC has been reported. This miRNA was found to inhibit tumor proliferation by suppression of mTOR [21]. Another study identified three miRNAs (miR-499, miR-135b and miR-205) as upregulated and five (miR-10b, miR-195, miR-30a-5p, miR-30a-3p and miR-21) as downregulated [22]. Apart from these prominent examples, many other miRNAs with EC-associated expression patterns have been identified. For example, Chung et al. [2] identified 30 miRNAs which were significantly deregulated in EC tumor samples, while Boren et al. [3] found 13, Weiguang et al. [5] found 23 and Cohn et al. [4] found 15 in early stage EC tumors and 18 in late stage tumors. Furthermore, Torres et al. [7] identified 21 dysregulated miRNAs from 122 samples, Zhang et al. [23] found 47 miRNAs from 73 samples, and Jurcevic et al. identified 138 miRNAs from 50 samples as differentially expressed between normal and tumor endometrium [18]. The wealth of EC-related miRNA data in the literature clearly demonstrate the need to collect information about all implicated miRNAs, since different studies may reflect different disease stages, patient subgroups, experimental methods, etc.

Studying the roles of miRNAs in a complex disease such as EC requires integration of data from several sources, including miRNA databases, miRNA target databases, gene annotation databases, expression data repositories and biomedical literature. To facilitate the integration of data and information from various sources, we developed the miREC (*miR*NAs in *E*ndometrial *C*ancer) database, which stores information about miRNAs experimentally shown to be aberrantly expressed in EC. The database also contains the genes that have been identified as targets of these miRNAs by prediction software or by experiments (or both). By storing the connections between EC-associated miRNAs and their computationally predicted and/or experimentally verified target genes, miREC provides a systematic overview of miRNA-regulation of processes involved in EC development. This information can be downloaded and visualized as a network using the Cytoscape [24] software. The miREC database is, to the best of our knowledge, the first systematic effort to integrate all available information about miRNAs and miRNA-targeted genes associated with endometrial cancer.

### Data sources

The miRNAs and their target genes were extracted from the studies of EC in *H. sapiens* which have been published to date [2-9,20,25-27]. The criteria used for miRNAs and genes to qualify for inclusion in miREC were the same as each author used for identifying significant deregulation (see Additional file 1: Table S1). Using a single criterion for all datasets was not feasible, since the studies were done using different experimental techniques (qRT-PCR or microarray), sample sizes, numbers of probes, etc., and since not all raw datasets were available. Applying the criteria resulted in extraction of 186 miRNAs and 576 genes shown experimentally to be deregulated in EC. *In silico* predicted and experimentally verified connections between miRNAs and target genes were also extracted to the extent that they appeared in the articles (275 predicted and 879 experimentally verified connections).

**Figure 1:**
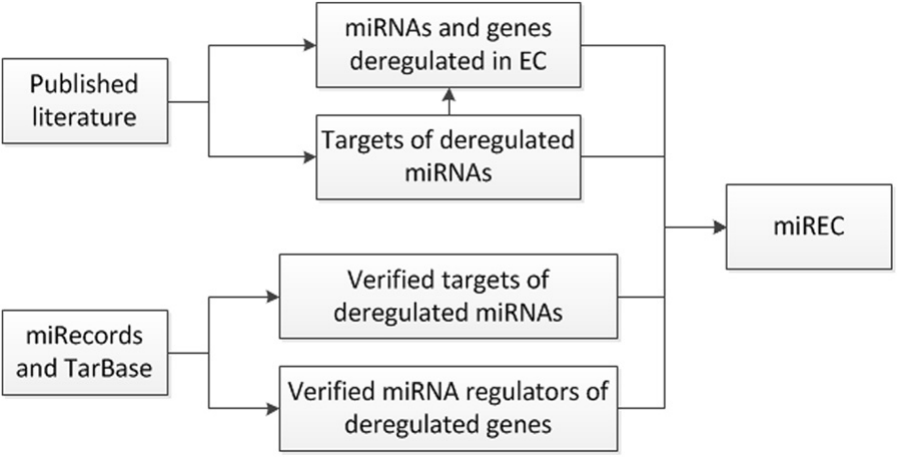
**Data extraction from literature and databases.** From the literature, miRNAs and genes that were reported as deregulated in EC were extracted, along with target genes (predicted and verified) of these miRNAs, as listed in the articles. For the miRNAs identified from the literature, the set of target genes was expanded with verified targets found in miRecords and TarBase, Similarly, for the genes identified from the literature, additional verified miRNA regulators were downloaded from miRecords and TarBase.

In addition to extracting data from the EC literature, information was also retrieved from the miRNA target databases miRecords and TarBase [28,29]. These databases contain experimentally verified regulator-target relationships between miRNAs and genes, and provide vital information to the understanding of the biological functions of miRNAs. MiRNAs and genes falling into the following two categories were extracted: (i) Genes that have been experimentally verified as targets of miRNAs that have been identified as deregulated in EC (344 genes); (ii) miRNAs that have been experimentally verified as targeting genes that have been identified as deregulated in EC (42 miRNAs). This raised the total number of miRNAs and target genes in miREC to 228 and 920, respectively. The miRecords version used was the 25th November 2010 update, and the TarBase version was 5.0. The scheme for information retrieval and storage in miREC is illustrated in Figure 1.

The information collected from the literature, miRecords and TarBase was complemented by information extracted from miRBase and the HUGO Gene Nomenclature Committee’s database (HGNC) [30,31], including gene names, IDs and symbols for genes and miRNAs, miRBase accession numbers and genome loci. The naming scheme for genes and miRNAs in miREC follows the HGNC nomenclature.

### Implementation

The miREC database was implemented as a relational database in mySQL on an Apache web server. A web interface was created using PHP to enable data input and searching in a user-friendly environment. The database is available online at http://www.mirecdb.org.

### Overview of database contents and structure

The current version of the database contains 228 miRNAs and 920 target genes. This may be contrasted with the number of human miRNAs in other data repositories such as microCosm, PhenomiR [32] and miR2Disease [33]. Compared to these databases, miREC is specialized towards miRNAs in endometrial cancer, which means that our database contains a smaller subset of all known miRNAs. This will allow the EC research community to find EC-specific information more easily. Of the entries in miREC, 186 miRNAs and 576 target genes come from published literature and the remaining ones from miRecords and TarBase. The aim of building the database was to facilitate a further understanding of EC development and provide a resource that will help identify miRNAs that can be evaluated for potential usage as markers for classification, diagnosis and prognosis. The information stored in the database includes:

- target genes and miRNAs;
- relations between miRNAs and their target genes;
- references to published scientific articles; and
- literature citations for genes, miRNAs and gene-miRNA relations.

For both target genes and miRNAs, the database stores the name, symbol, HGNC ID, organism, chromosome positions, type of deregulation in EC (up- or downregulated, or unspecified), and verification status of the deregulation. For miRNAs, the miRBase accession number is also stored. The citation tables keep track of the literature sources where information about miRNAs, target genes, and miRNA-target gene relations was found. This allows the full article references to be displayed on the information pages for each miRNA and target gene entry. The information in miRNA and target gene entries is complemented with links to other databases.

### Querying the database

**Figure 2:**
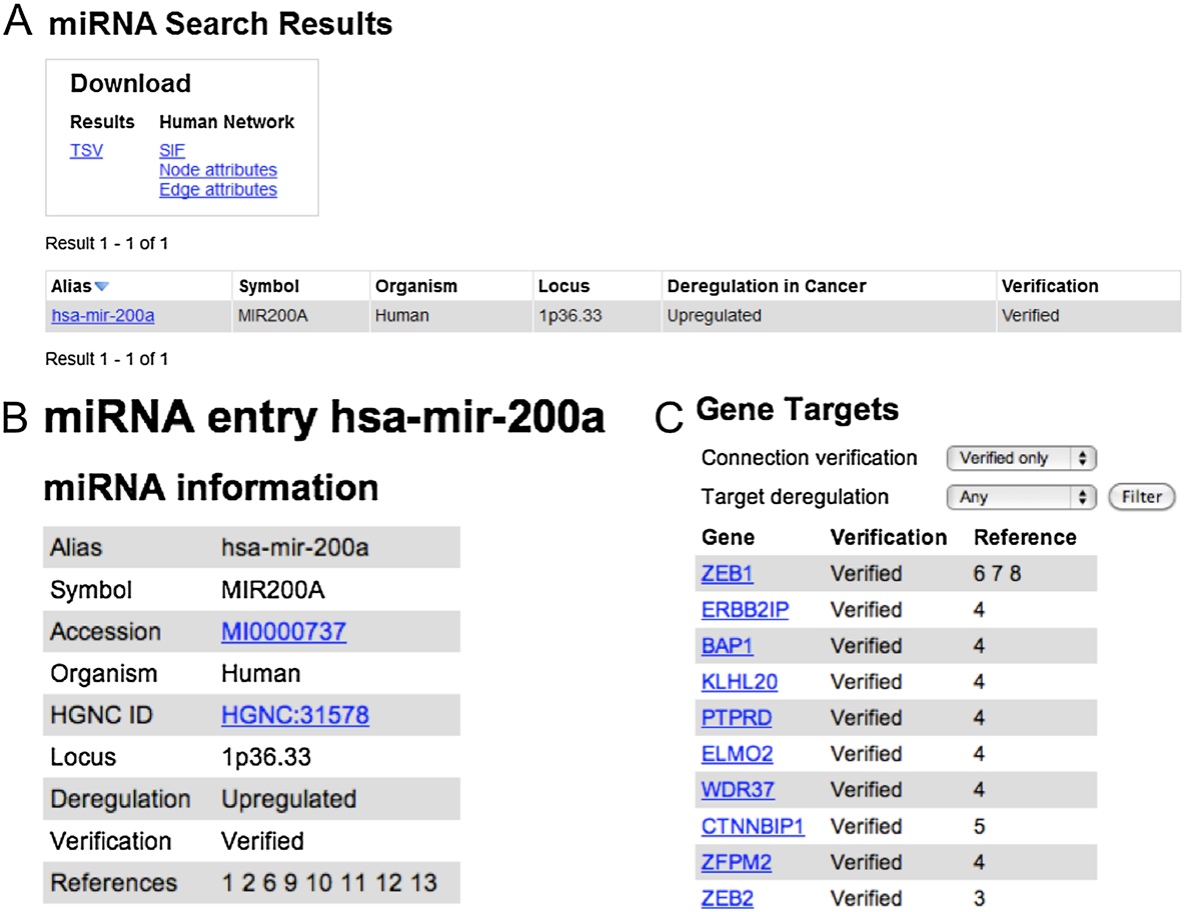
**Result from Quick Search on hsa-mir-200a. (A)** Results of a quick search for the miRNA hsa-mir-200a. **(B)** More detailed page with information relating to the same microRNA. The contents of this page are divided into three sections: (1) *miRNA information*, including alias, symbol, accession number, organism, HGNC ID, chromosome location, form of deregulation, deregulation verification status and references; (2) *Gene Targets*, showing target genes, verification status of each miRNA-target association, and references; (3) *Articles,* showing a detailed list of references, including links to PubMed entries (not shown in the figure).

A wide range of search options is available via the web interface, allowing the user to search the database for genes, miRNAs and regulatory relationships. The main page presents a Quick Search option, while advanced search options can be found through the menu. Quick search has two options: search by gene and search by miRNA. Figure 2 shows an example quick search for miRNAs and how the results are displayed as a table in the web browser (Figure 2A). This table may contain a number of different miRNAs or genes, as all database entries that contain the query text in the name, symbol or accession number will be retrieved. By clicking the miRNA name in the result list, a page with more detailed information about the miRNA will be displayed (Figure 2B), including links to external databases (miRBase and HGNC) with additional annotation, the observed form of deregulation in cancer (up- or downregulated) and the verification status of the deregulation (experimentally verified or not), a list of predicted and/or experimentally verified gene targets (Figure 2C), and a list of publications serving as references for the information with links to their PubMed entries.

From the page shown in Figure 2C it is possible to go to the information page of each target gene. Thereby the user can get information about other miRNAs that regulate the same target gene. The lists of target genes and regulating miRNAs can be filtered by using the verification and deregulation options, so that, for example, only downregulated target genes are shown. Many more options are available in the advanced search forms. For example, it is possible to search for all genes targeted by a specific miRNA, or to search for all miRNAs targeting a specific gene. Many different searches are possible by setting combinations of parameter values. For example, specifying hsa-mir-106a as regulator, setting the option “Number of regulators” to “3” and the option “Verification of regulator connections” to “Verified”, the result would only include genes which have been experimentally shown to be regulated by hsa-mir-106a and at least two other miRNAs.

**Figure 3:**
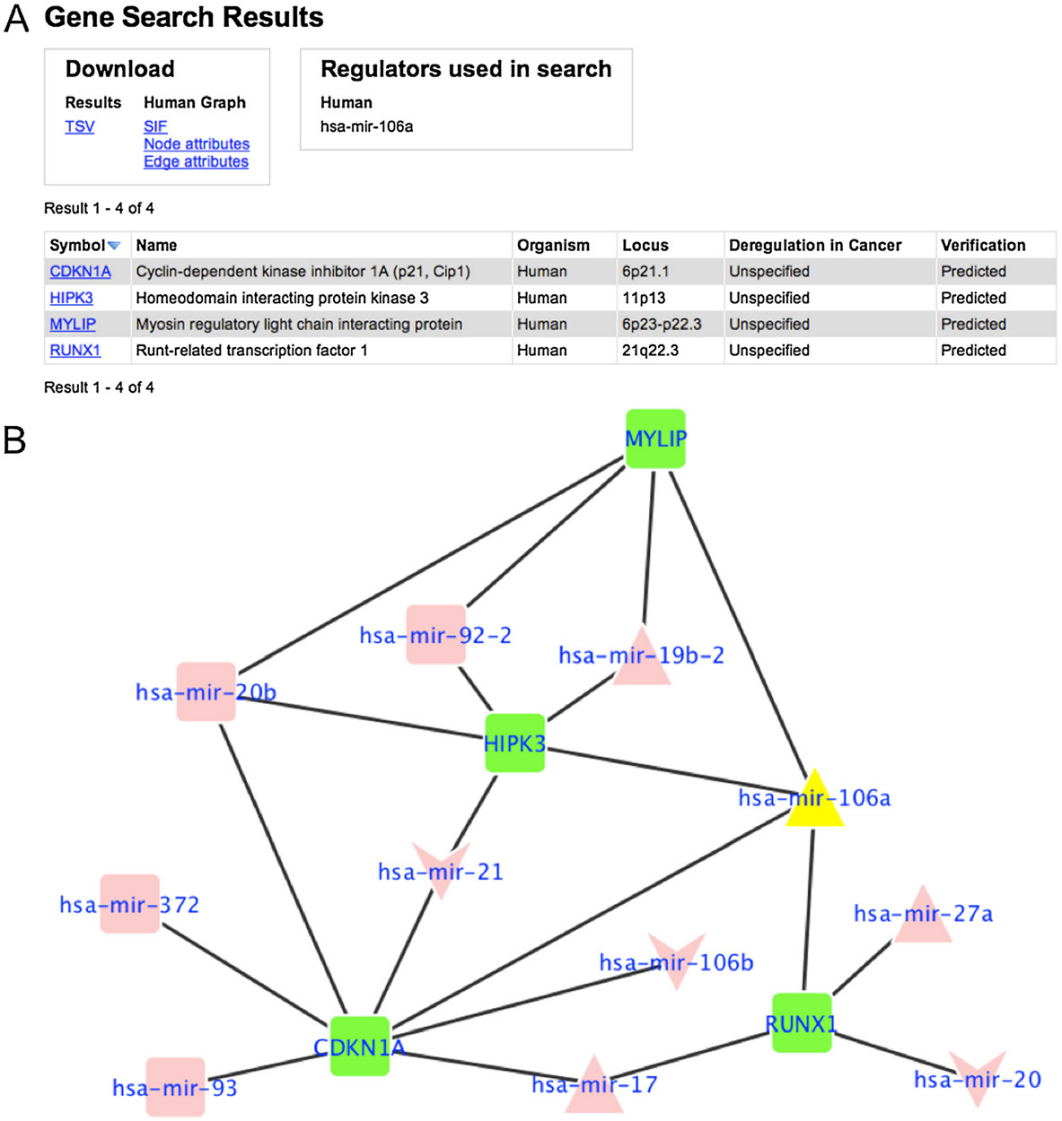
**Demonstration of advanced search. (A)** Results from a search for genes with *hsa-mir-106a* as miRNA regulator (without restrictions on verification status or number of regulators). **(B)** Network generated from the same search, showing *hsa-mir-106a* (yellow triangle), four of its target genes (green nodes) and other miRNAs targeting these genes (pink nodes). Triangles indicate up-regulation in cancer, V shapes indicate down-regulation and squares indicate that there is no experimental data on expression in EC. Edges indicate gene-miRNA relationships, with thick black edges indicating verified regulation. The network can be downloaded using the SIF hyperlink under the heading Human Graph above the results table in Figure 3A.

The results of a search for all genes regulated by hsa-miR-106a are shown in Figure 3A. This miRNA has been identified as dysregulated in EC in three studies [2,3,18]. To visualize the results, a gene-miRNA interaction network can be downloaded using the links above the results table. The network is in the Simple Interaction Format (SIF) and can be imported and viewed in the free Cytoscape software [24]. Supplementary node and edge attribute files can also be downloaded and imported into Cytoscape to enrich the visualization with information for all genes, miRNAs and connections. Figure 3B shows a network generated from the search results of Figure 3A. Such visualizations integrate different pieces of information regarding the genes/miRNAs of interest and make it easier to formulate research questions and hypotheses based on the available information, as exemplified in the next chapter.

### Identifying miRNA clusters

The network in Figure 3B shows that hsa-mir-106a along with three other miRNAs (hsa-mir-17; hsa-mir-19b-2 and hsa-mir-27a) have verified connections (thick edges) to a number of genes. Two of these genes are HIPK3 and MYLIP, which are regulated by hsa-mir-106a and hsa-mir-19b-2. Two other genes, RUNX1 and CDKN1A, are regulated by hsa-mir-106a and hsa-mir-17. A literature search showed that two of these miRNAs, namely hsa-mir-106a and hsa-mir-19b-2, belong to the same miRNA cluster on human chromosome X. Furthermore, this cluster is a paralog of the mir17 cluster on chromosome 13, in which hsa-mir-17 is located. Specifically, hsa-mir-106a is a homolog to hsa-mir-17 [34]. This demonstrates how a database query in miREC, followed by analysis of the resulting network, leads to identification of a functional relationship between miRNAs that belong to the same or related clusters in the genome. In this particular example, the part of the network analyzed in this paragraph corresponds to information gathered from four different articles, which is easily identifiable in the visualization.

### Clustered miRNAs share target genes

**Figure 4:**
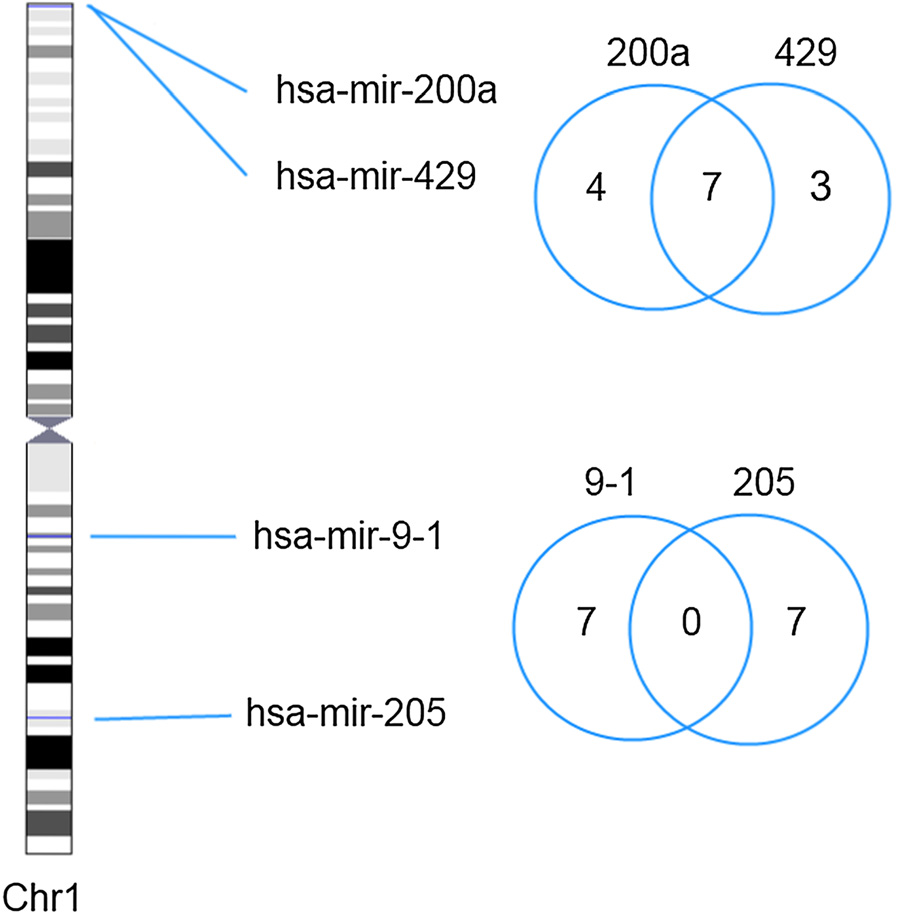
**Comparison of verified target gene sets for pairs of clustered miRNAs versus pairs of distant miRNAs.** The two miRNAs hsa-mir-200a and hsa-mir-429 are located <10,000 bp from each other on chromosome 1 and thus belong to the same cluster. Two other miRNAs, hsa-mir-9-1 and hsa-mir-205, are not within the distance limit and thus considered distant. While hsa-mir-200a (with 11 targets) and hsa-mir-429 (with 10 targets) have seven targets in common, the distant pair hsa-mir-9-1 and hsa-mir-205 (with seven targets each) do not have any target genes in common.

The miREC database can also be used to analyze whether there is a general tendency for miRNAs located in the same clusters to share target genes. To investigate this, the number of common target genes for pairs of miRNAs located in the same cluster was compared to the number of common targets for pairs of miRNAs not located in the same cluster (Figure 4). A search for all miRNAs in miREC with verified target genes identified 169 miRNAs with a total of 792 targets (average 4.69). Using a distance limit of 10 000 nucleotides, 74 of these miRNAs were found to reside in 30 clusters, of which 21 clusters with two miRNAs each, six with three miRNAs each, two with four miRNAs each and one cluster with six miRNAs. Thus, we could form 66 pairs of miRNAs where both were located in the same cluster. The remaining 95 miRNAs were labeled as “distant miRNAs”. The average number of target genes was 10.3 and 12.5 for clustered and distant miRNAs, respectively.

The number of shared targets was then counted for the 66 pairs of miRNAs located in the same clusters and for all 4,465 pairs of distant miRNAs. It was found that the 66 clustered pairs of miRNAs had 175 common targets while the pairs of distant miRNAs had 947 common targets, i.e. an average of 2.65 versus an average of 0.21. Seventeen of the pairs of distant miRNAs were formed between duplicates (e.g. hsa-mir-1-1 and hsa-mir-1-2), which have identical sequences and therefore obviously identical sets of target genes. When these duplicate pairs, which had 692 target genes in common (40.7 on average) were removed from the analysis, the average number of shared targets for pairs of distant miRNAs dropped to 0.06.

For the miRNA cluster data analyzed in this study, clustered pairs of miRNAs shared more than 12 times as many target genes as pairs of distant miRNAs. This indicates that the clustering of miRNAs in genomes throughout evolution is important for the regulation of transcription. It seems reasonable that clustered miRNAs can more easily be co-regulated and thus function as a regulatory unit, thereby coordinately affecting the activity of the biological processes in which their shared target genes are involved. Interestingly, a recent study by Sass *et al.* [35] provides evidence of co-expressed miRNAs (including clustered miRNAs) targeting different components in the same protein complex. Other authors have shown that the genomic organization of some miRNA clusters have been preserved over millions of years of evolution, which indicates a functional advantage of this arrangement [36]. That clustering facilitates coordinated regulation of components, both in protein complexes and in pathways, may be the explanation for the tendency for this genomic organization to be preserved.

### Enabling cancer hallmarks in endometrial cancer through miRNA dysregulation

**Figure 5:**
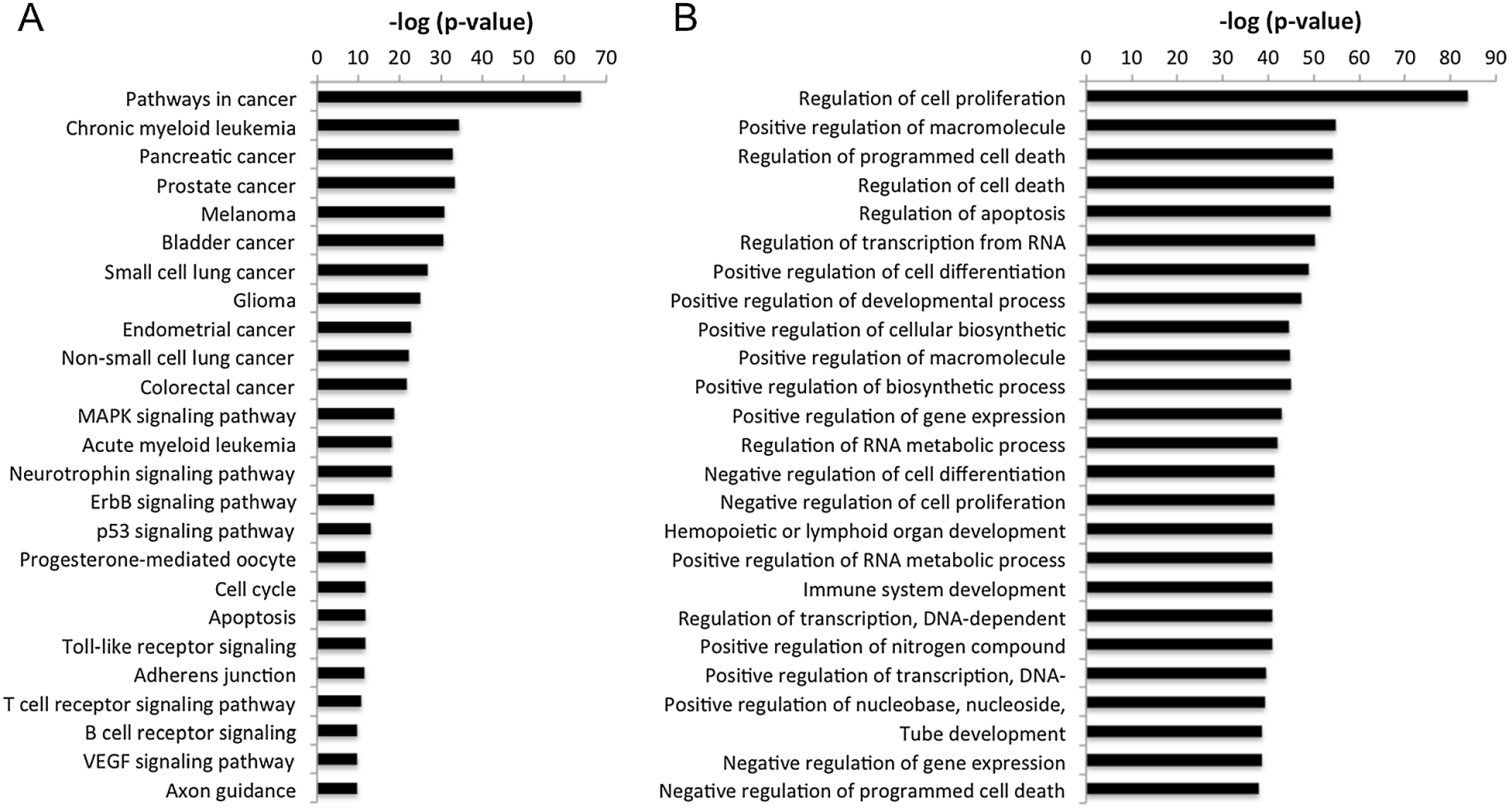
**Significantly enriched gene annotation among the verified target genes.** Significance level was set to 0.05 for Benjamini-Hochberg-corrected p-values. Panel **A**: Enriched KEGG pathways. Panel **B**: Enriched biological processes.

**Figure 6:**
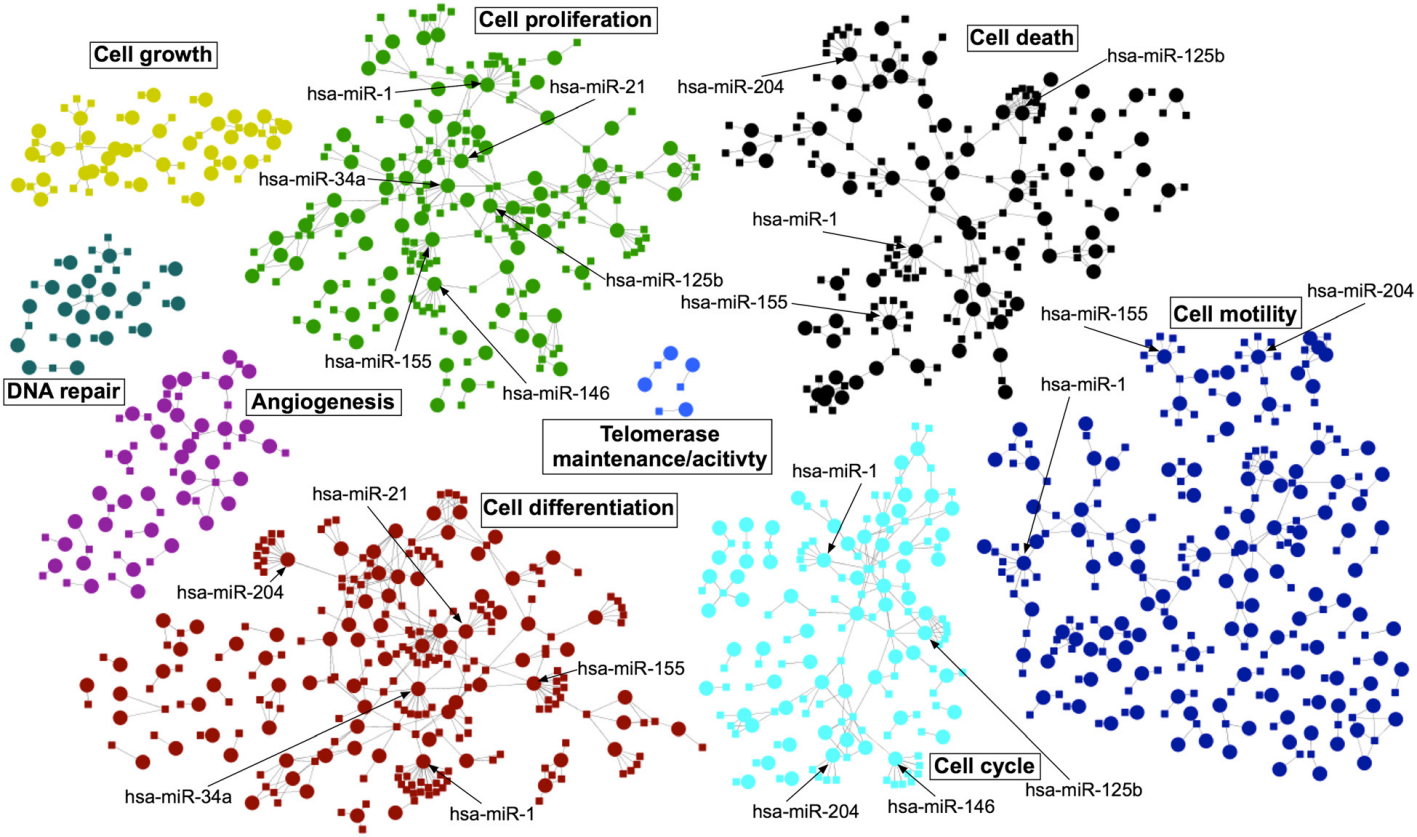
**Network of dysregulated miRNAs and their target genes annotated with biological processes.** Genes are indicated by small squares and miRNAs are large circles. Processes are colored as followed: Cell growth (yellow), cell proliferation (green), cell death (black), DNA repair (dark green), telomerase activity and telomere maintenance (light blue), angiogenesis (purple), cell differentiation (red), cell cycle (teal) and cell adhesion/motility (dark blue).

It is recognized that, in order for tumors to develop, cells must acquire cellular characteristics that are very different from those of healthy cells. These characteristics are referred to as cancer hallmarks and give tumor cells an evolutionary advantage over normal cells e.g. in terms of growth and proliferation [37]. Therefore, to understand tumorigenesis, the acquisition of these cancer hallmarks must be studied in different tumors. Since individual miRNAs have potentially hundreds of target genes, miRNA dysregulation will have a profound effect on the regulation of the cellular machinery and contribute to enabling cancer hallmarks. To study this for EC, all miRNAs experimentally shown to be dysregulated in endometrial cancer were downloaded from miREC, along with their experimentally verified target genes (93 miRNAs and 472 genes). As an example of how to study the biological annotation for the genes in miREC, the gene list was submitted to DAVID and annotation enrichment analysis was carried out by inspecting terms for pathways and biological processes (Figure 5). The results clearly demonstrate a significant enrichment of cancer-specific pathways and pathways frequently altered in cancer (Figure 5A), such as MAPK, ErbB, p53, Toll-like receptor and VEGF signaling pathways. TFG-β, mTOR and Wnt signalling pathways were also enriched (data not shown). Several significant biological processes related to cancer hallmarks were detected, including regulation of cell proliferation, cell death, metabolic processes, transcription, differentiation and immune system development (Figure 5B). Enrichment was also seen for cell adhesion, cell motion and angiogenesis GO terms (data not shown). To visualize the miRNA-mediated regulation of these genes, a network of the genes and miRNAs was also downloaded from miREC (Figure 6). The genes were annotated with terms from the biological process and molecular function hierarchies of Gene Ontology. To reduce the granularity of terms and more effectively highlight cancer hallmark genes in the network, the following procedure was used. First, annotation terms assigned to the genes were downloaded, including the following terms and their respective child terms: cell proliferation, cell growth, cell death, angiogenesis, cell cycle, cell differentiation, telomerase activity, telomere maintenance, cell adhesion, cell motility and DNA repair. Second, all child terms were replaced with their parent term from the first step. Third, the terms of the genes were propagated to their regulator miRNAs. The resulting network (Figure 6) contains 295 genes and 93 miRNAs. Since many genes and miRNAs had multiple terms, a node in the network was created for every term of a given gene or miRNA. The resulting network highlights the importance of key miRNAs, such as the central location of hsa-miR-34a in the cell proliferation and differentiation sub-networks [38,39].

**Figure 7:**
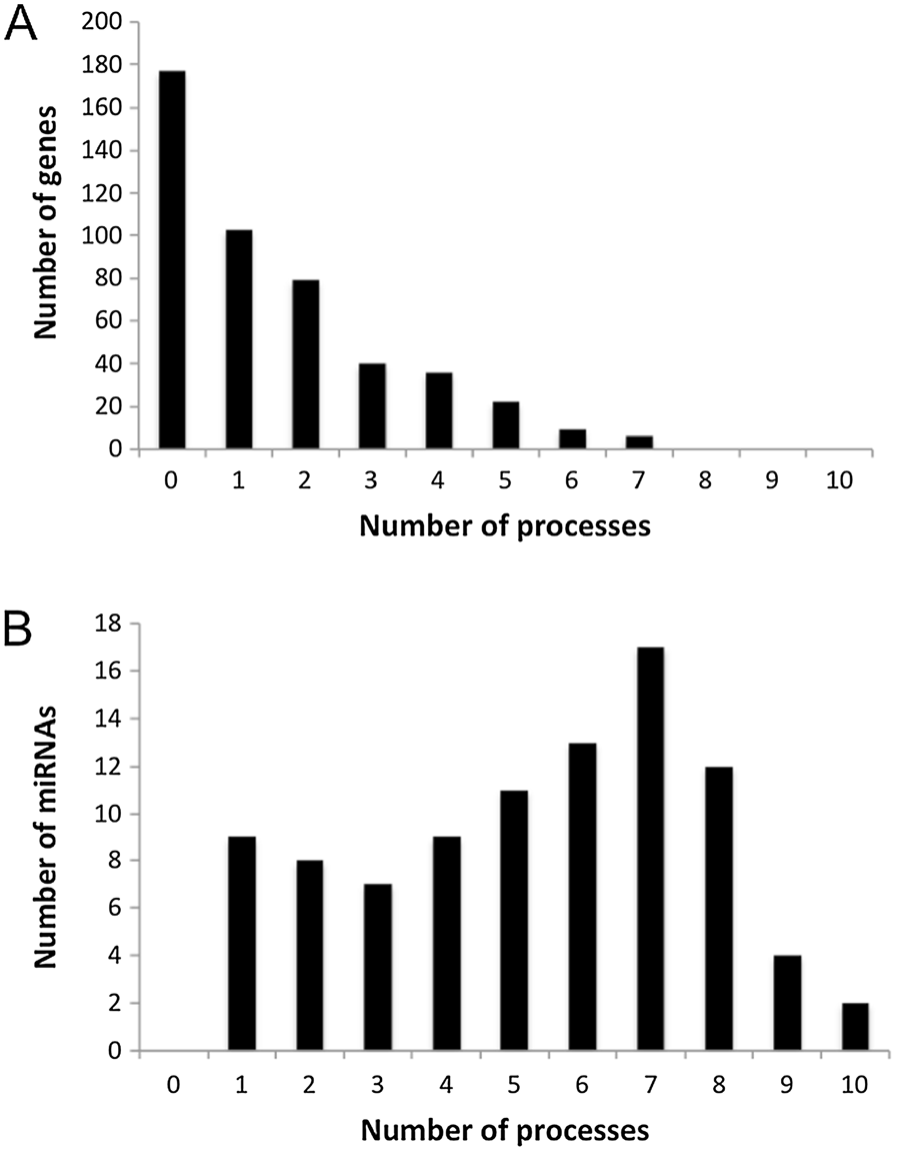
**Number of biological processes per gene and miRNA.** Panel **A**: Number of processes associated with genes. Panel **B**: Number of processes associated with miRNAs.

This network allows detailed analysis of regulatory mechanisms by miRNAs involved in endometrial cancer hallmarks (Additional file 2). As an example, hsa-miR-125b, hsa-miR-146 and hsa-miR-27b regulate three genes involved in telomerase activity and telomere maintenance (HIST1H4A, POLE2 and PPARG). HIST1H4A is involved in telomere maintenance and is regulated by hsa-miR-125b (experimentally supported). Although hsa-miR-125b is downregulated in EC tumors, the expression of HIST1H4A in EC has not been reported in the literature. By suppressing hsa-miR-125b, HIST1H4A could be upregulated allowing the tumor to stabilize its telomeres. Furthermore, PPARG is a negative regulator of telomerase activity and is itself regulated by hsa-miR-27b, which is upregulated in EC. PPARG upregulation at the mRNA level has been reported in the literature [40,41], but the protein is downregulated in tumors [41]. Thus hsa-miR-27b may prevent translation of PPARG mRNA and allow tumors to prevent suppression of telomerase; further supporting telomere stabilization to enable replicative immortality. However, POLE2 is also involved in telomere maintenance and is regulated by hsa-miR-146, which is upregulated in EC. This appears to oppose replicative immortality, which reveals the complexity of miRNA-mediated regulation. This complexity is further highlighted by the fact that the miRNAs in Figure 6 regulate five biological processes on average. The number of processes per gene and miRNA is shown in Figure 7. The question of how EC tumors enable immortality clearly presents a potential future direction in EC research. The miREC database allows researchers to identify both miRNAs and genes of interest based on high-confidence data.

## Conclusions

The miREC database was created to provide information about miRNAs and genes that are potentially involved in the development of endometrial cancer. There are several general databases that contain information about miRNAs and their target genes, such as miRecords. In contrast, miREC focuses on data that is specific for EC, which makes it easier for researchers to derive disease-specific information, e.g. miRNA-target interaction networks including only those miRNAs that are deregulated in EC. The wide range of search functions in miREC makes it possible to find EC-associated miRNAs from many different starting points, such as target genes, type of deregulation, genomic location, etc.

By analyzing data from the miREC database, it was found that the number of common target genes was significantly higher for pairs of miRNAs located in the same cluster compared to random pairs of distant miRNAs. Recent studies have shown that clustered miRNAs are often co-expressed and often transcribed as polycistrons [36,42], and it has been suggested that this supports coordinated miRNA regulation of protein complexes and target pathways [36,43]. The result from the analysis on common targets of clustered miRNAs presented here also supports this hypothesis. Furthermore, since miRNAs regulate genes involved in several cancer hallmarks, the dysregulation of miRNAs will allow tumors to acquire several characteristics necessary for tumorigenesis.

The database is very general and contains data from all published studies found in the literature survey. Using the search functions subsets of genes and miRNAs of interest can be downloaded for analysis to test hypotheses of interest to the user. It is then important to apply suitable quality control, since the data was collected from studies using different experimental techniques, sample sizes, numbers of probes, etc. Furthermore, biopsies from which RNA is extracted may contain heterogeneous cell populations with respect to cell type, tumor stage, etc. This may confound statistical analysis and interfere with measuring gene expression in tumor cells [44]. Since the aim of the literature survey was to collect all studies of gene and miRNA deregulation in EC, the risk of confounded results was not investigated further.

The miRNA data will in future versions of the database be complemented with additional layers of disease information, such as gene and protein expression, genome alterations and epigenetic patterns, to establish a more general platform for EC research. The database is designed to allow organism-specific information to be added, which means that information obtained from animal models can be used to complement the information from human. In future updates of the miREC database, results derived from a rat model of EC [45] will be added to enable comparative mapping of miRNAs. Due to the high degree of conservation between human and rat, the information from the rat experiments can easily be extrapolated to human. Finally, results from the rat model experiments will be used in our efforts to derive a miRNA expression signature for EC diagnosis in humans.

## Availability and requirements

**Project name:** miREC database

**Project home page:**http://www.mirecdb.org

**Operating system:** Any

**Programming language:** None

**Other requirements:** None

**License:** GNU GPL

**Any restrictions to use by non-academics:** None

## Additional files

Additional file 1: Table S1. Curated Studies: Table containing published studies in the literature from which data in the miREC database have been curated. Additional file 2:
**Annotation network:** **Network created in Cytoscape using miRNAs and genes downloaded from miREC version 2 and Gene Ontology annotation.**
